# Supplementary material for: Quantification of droplet and contact transmission risks among elementary school students based on network analyses using video-recorded data
Source: PLoS One. 2025 Feb 12;20(2):e0313364. doi: 10.1371/journal.pone.0313364 (PMC11819611; doi:10.1371/journal.pone.0313364)
Supplement: Attachment — Submitted filename: ploson review.docx [file pone.0313364.s014.docx]

First of all, I would like to state that this publication is a very scientific, technical and interesting research.

The text of the article has been uploaded to the system twice. There are referencing deficiencies, typographical errors, omissions. The latest version needs to be uploaded.

Line 175-176 also has two communication behavior headings.

It will be of interest to the reader to give brief information in the method section about what kind of research and analysis is the micro-simulation.

It should be noted that the situation of the teacher factor in the classroom was not analyzed in the study, and there was no cleaning in the classroom.

From the contacted materials, it should be indicated whether the backpack is kept in cabinets.

If there is information about how long the cases wore a mask, it should be specified.

It should be noted that the image is not taken in hand washing, toilets and faucets.

In Figure 6, the word numver should be corrected as number.

What is Heatmap? How is it interpreted and what does it mean? A brief description of this topic informs the reader and attracts attention.

Thank you. Congratulations.
