# Supplementary material for: Quantification of droplet and contact transmission risks among elementary school students based on network analyses using video-recorded data
Source: PLoS One. 2025 Feb 12;20(2):e0313364. doi: 10.1371/journal.pone.0313364 (PMC11819611; doi:10.1371/journal.pone.0313364)
Supplement: Attachment — Submitted filename: Response to Reviewers.docx [file pone.0313364.s015.docx]

Response to reviewers’ comments

Reviewer 1, Concern 1: The language of the article is not perfect, ‎containing ‎multiple grammar and verbal errors throughout the text, which hampers ‎perception of the ‎work. I recommend that the authors should carefully revise the text for ‎the language style ‎and errors, for which purpose they are advised to consult those who ‎have expertise in ‎writing scientific articles in English.‎

Author response: Thank you for pointing out the grammar and verbal errors. We have revised the manuscript using a professional English proofreading service.

Author action: The results of the English proofreading have been applied throughout the entire manuscript.

Reviewer 1, Concern 2: The introduction of the manuscript should be analyzed the critical gap in the ‎literature ‎and how the present study mitigates the gap.

Author response: To estimate virus transmission in elementary schools, it is essential to obtain behavioral data from students that detail who was involved, when, where, and what activities were performed, particularly concerning droplet and contact transmissions. In previous studies, questionnaires and wearable sensor devices were used for communication behavior, and swab sampling was employed for contact behavior. However, these methods have been insufficient in capturing detailed information about the processes and actions of each student that contribute to pathogen transmission. In this study, we addressed this gap by simultaneously collecting behavioral data related to both droplet and contact transmission through video recording. Using video recording, we were able to obtain the processes and actions of each individual student. This approach enables the identification of potential factors contributing to both droplet and contact transmission.

Author action: We have added a "Related work" section to the manuscript, where we have described the critical gap in the literature and how the present study mitigates this gap.

Reviewer 1, Concern 3: The objectives are not clearly ‎written ‎and mentioned.‎

Author response: Thank you for your suggestion to clarify the objectives, which has helped improve the quality of the manuscript. To estimate virus transmission risk in elementary schools, it is essential to obtain behavioral data from students that detail who was involved, when, where, and what activities were performed, particularly concerning droplet and contact transmissions. However, these methods have been insufficient in capturing information about the processes and actions of each student that contribute to pathogen transmission. Therefore, in this study, we aim to accurately assess the comprehensive pathogen transmission risk within classroom environments in elementary schools by using video-recorded data to observe students' behaviors in real-time.

Author action: We have included the clarified objective of this study at the beginning of the 4th paragraph in the "Introduction" section.

Reviewer 1, Concern 4: The abstract and conclusion sections should be improved to show the main ‎research ‎findings.‎

Author response: We appreciate the opportunity to better highlight the main research findings. We have revised the abstract and the conclusion section to clearly present the key results of our study.

Author action: We have added statements to the "Abstract" and "Conclusion" sections to emphasize the main findings.

Reviewer 1, Concern 5: The authors should put more efforts on the discussion with the help of latest ‎and ‎pertinent references.‎

Author response: Thank you for your suggestion to improve the quality of the manuscript. Using the latest and pertinent references, we discussed how the structure of the communication network aligns with findings from previous studies. In previous swab sampling surveys, the possibility of virus transmission via shared items, such as doors and faucets, has been investigated. This study revealed that belongings owned by others, such as desks and shirts, have the potential to contribute to virus transmission by contact network analysis and micro-simulation. In addition, shared items with high contact frequency and high centrality metrics in the network, which were not evaluated in swab sampling surveys, exhibited substantial viral adherence.

Author action:

- Regarding the communication network, we included this discussion in the 5th paragraph of the "Characteristics of communication between students" subsection under "Communication behavior" in the "Results and Discussion" section.
- Regarding virus transmission potential items, we added this discussion at the end of the 2nd paragraph of the "Characteristics of contact items" subsection under "Contact behavior" in the "Results and Discussion" section.
- Regarding the comparison of micro-simulation results with previous studies, we included this discussion in the 2nd paragraph of the "Analysis of contact infection with micro-simulation" subsection under "Contact behavior" in the "Results and Discussion" section.

Reviewer 2, Concern 1: Line 175-176 also has two communication behavior headings.

Author response: Thank you for pointing out the duplication of headings. One of the headings has been changed to "Characteristics of communication between students" to resolve the issue.

Author action: We updated the manuscript by modifying the heading under the “Contact behavior” of “Results and Discussion”.

Reviewer 2, Concern 2: It will be of interest to the reader to give brief information in the method section about what kind of research and analysis is the micro-simulation.

Author response: Thank you for your suggestion to improve the quality of the manuscript. Micro-simulation is a simulation technique that predicts dynamic changes in societies, economies, cities, and organizations by replicating individual behaviors based on actual behavioral data using probabilistic models. In our study, we used it to evaluate scenarios where a virus-carrying student was present based on the observed contact behaviors.

Author action: We added this information at the beginning of the "Micro-simulation" subsection under the "Method" section.

Reviewer 2, Concern 3: It should be noted that the situation of the teacher factor in the classroom was not analyzed in the study, and there was no cleaning in the classroom.

Author response: Thank you for pointing out the teacher factor and cleaning in the classroom. We believe that the influence of teachers on virus transmission exists. In this study, we conducted analyses excluding teachers; however, it is important to consider teacher factors in future research. Additionally, cleaning was not performed during the period when the micro-simulation was conducted.

Author action: We added a 3rd paragraph to the "Limitation" section to address the teacher factor. Additionally, we included a statement regarding the lack of cleaning in the classroom in the 2nd paragraph of the "Analysis of contact infection with micro-simulation" subsection under "Contact behavior" in the "Results and Discussion" section.

Reviewer 2, Concern 4: From the contacted materials, it should be indicated whether the backpack is kept in cabinets.

Author response: Thank you for this valuable suggestion. School bags are usually kept in lockers at the back of the classroom. Thus, it is rare to contact them.

Author action: We have addressed the location of school bags in the 2nd paragraph of the "Characteristics of contact items" subsection under "Contact behavior" in the "Results and Discussion" section.

Reviewer 2, Concern 5: If there is information about how long the cases wore a mask, it should be specified.

Author response: We appreciate your comment regarding the wearing of masks by students. The students were always wearing masks during the observation period.

Author action: We have added a statement about the mask-wearing situation to the "Limitation" section.

Reviewer 2, Concern 6: It should be noted that the image is not taken in hand washing, toilets and faucets.

Author response: Thank you for pointing out that images related to hand washing, toilets, and faucets are not included.

Author action: We have added this note to the 2nd paragraph of the "Data collection/ethics statement and privacy" subsection under the "Method" section.

Reviewer 2, Concern 7: In Figure 6, the word numver should be corrected as number.

Author response: We apologize for the typographical error. The word "numver" in Figure 6 has been corrected to "number."

Author action: We have replaced Figure 6 with the corrected version addressing the typographical error.

Reviewer 2, Concern 8: What is Heatmap? How is it interpreted and what does it mean? A brief description of this topic informs the reader and attracts attention.

Author response: We apologize for the lack of explanation. We have added details about the indicators represented by the colors in the heatmap and their meanings.

Author action: We included explanations of the heatmaps in Figures 9 and 11 in the 2nd paragraph of the "Network analysis" subsection under "Contact behavior" and the 3rd paragraph of the "Analysis of contact infection with micro-simulation" subsection, respectively, in the "Results and Discussion" section.
